# Corroboration of cross-reactivity between *Mycobacterium leprae* and hosts’ salivary and cutaneous proteins: A hope for prognostic biomarkers for the pathogenesis of reactions in leprosy

**DOI:** 10.3389/fmicb.2022.1075053

**Published:** 2022-12-06

**Authors:** Vinay Kumar Pathak, Itu Singh, Shoor Vir Singh, Utpal Sengupta

**Affiliations:** ^1^Stanley Browne Laboratory, The Leprosy Mission Community Hospital, New Delhi, India; ^2^Department of Biotechnology, GLA University, Mathura, Uttar Pradesh, India

**Keywords:** leprosy, cross-reactivity, molecular mimicry, reactions, biomarkers

## Abstract

**Introduction:**

Immunological reactions are frequent complications that may occur either before, during, or after treatment and affect 30–50% of leprosy patients. The presence of autoantibodies like rheumatoid factor, antinuclear factor, and antibodies to host collagen, keratin, actin, myosin, endothelial cells, and myelin basic protein (MBP) has been earlier reported in leprosy patients. The purpose of this study was to identify cross-reactive proteins in clinical samples such as saliva and slit skin scrapings (SSS) of leprosy patients which could be utilised as prognostic biomarkers for Type 1 Reaction (T1R) in leprosy.

**Method:**

A total of 10 leprosy patients in T1R and 5 healthy volunteers were recruited. The protein was extracted from their SSS and saliva samples, thereafter, isoelectric focusing (IEF) and two-dimensional PAGE were performed to analyse the proteins. Furthermore, the cross-reactivity was identified by western blotting host proteins in gel against purified IgG from Mycobacterium leprae soluble antigen (MLSA)- hyperimmunized rabbit sera, thereafter, cross-reactive proteins were identified by MS/MS. The cross-reactive host proteins were analysed for homologous bacterial proteins and B cell epitopes (BCEs) were predicted by using bioinformatic tools.

**Results:**

A total of five spots of salivary proteins namely S100-A9, 35.3 kDa, and 41.5 kDa proteins, Serpin peptidase inhibitor (clade A), Cystatin SA-III, and four spots of SSS namely 41.4 kDa protein, Alpha-1 antitrypsin, vimentin, and keratin 1, were identified as cross-reactive. Further, a total of 22 BCEs of cross-reactive host proteins were predicted and visualised.

**Discussion:**

This data provides strong evidence of cross-reactivity/molecular mimicry between host and pathogen in leprosy patients with reaction. These BCEs of cross-reactive proteins could be further studied to predict reactions and may be utilised as an early diagnostic biomarker for T1R in leprosy.

## Introduction

Microbial pathogens that have persistently long incubation periods may have sophisticated strategies to subvert the cellular functions of the host, which could be one of the reasons to escape the defence mechanism of the host. Mycobacteria are known to modulate host immune responses, both in humans and experimental animals (Shoenfeld and Isenberg, 1988). In addition to the immune response to the pathogen, mycobacterial infections have been also shown to evoke an autoimmune response (Rook, 1988; Sher et al., 1978).

Reactions are immunological complications that occur either before, during, or after treatment and can affect 30–50% of leprosy patients (Scollard et al., 1994; Scollard et al., 2006). Type 1 reaction (T1R) is characterised by acute inflammation of skin lesions and/or nerves and is a major cause of neuritis, often leading to nerve damage resulting in deformities of mainly the hands and feet. The immune response is characteristic of a delayed-type hypersensitivity (DTH) reaction with an influx of peripheral blood lymphocytes demonstrating an increased reactivity to *Mycobacterium leprae* antigens in a lymphocyte transformation test (Bjune et al., 1976). Although several mechanisms have been proposed for the pathogenesis of immunological reactions in leprosy, the intricate mechanisms are still not well understood, and the present study suggests an additional possible mechanism mediated by autoimmune factors. Galán suggested that antigenic/molecular mimicry between host and pathogen is a way to escape detection and destruction of the pathogen by host immunity (Galán, 2000). It might be one of the reasons for escaping the immune surveillance of the host by *M. leprae* leading to a long period of incubation before the onset of clinical manifestation.

Infection with *M. leprae* may induce considerable changes in the humoral immune system, often associated with the autoimmune syndrome. Leprosy, more specifically at the LL pole, is frequently associated with a range of auto-antibody responses, both organ-specific (directed against the thyroid, nerve, testis, and gastric mucosa) and non-specific, such as rheumatoid factor (Petchclai et al., 1973), antineutrophil cytoplasmic autoantibodies (ANCA) (Medina et al., 1998), antinuclear antibodies (ANA) (Miller et al., 1987), and anti-phospholipids (Arvieux and Hachulla, 2002) as well as cryoglobulins (Bonomo and Dammacco, 1971). Other auto-antibodies, like anti-mitochondrial (Guedes Barbosa et al., 1996), anti-actin, anti-myosin (Kroumpouzos et al., 1993), and anti-endothelial cell antibodies (Dugué et al., 2004), have also been reported in patients with leprosy. A conserved mycobacterial membrane protein and mycobacterial ferredoxin-NADP-reductase have been reported to mimic human myelin P0 (a protein that assists in compacting myelin) and it has been suggested that this phenomenon might be responsible for demyelination (Vardhini et al., 2004). Recently, our team has reported a significant correlation of eight mimicking B cell epitopes (BCEs) and five mimicking T cell epitopes (TCEs) between *M. leprae* and host components with the inflammatory episodes of T1R in leprosy (Pathak et al., 2021).

Antigenic mimicry may be one of the reasons for avoiding *M. leprae* immune surveillance resulting in a long incubation period before disease onset. Epitope similarity may promote the survival of *M*. *leprae* in human hosts if the antigen is not recognised as a “foreign”. However, immune recognition of *M*. *leprae* antigens that mimic host antigens will trigger autoimmune responses against the host’s antigens. Antigenic mimicry between pathogen and host may play a role in the pathogenesis of leprosy and be one of the reasons for immunological complications. The findings above suggest that molecular mimicry might be involved in the pathogenesis of leprosy as well as the onset of immunological reactions. Therefore, studies to examine cross-reactive proteins in different types of clinical samples are required to expand knowledge about the role of molecular mimicry in the pathogenesis of leprosy. Hence, the present study was carried out to identify the presence of cross-reactive proteins in clinical samples such as saliva and slit skin scrapings (SSS) of leprosy patients with T1R, which may result in the identification of novel proteins/biomarkers that might be useful in predicting reactions in leprosy. Early diagnosis of T1R is very much needed and in demand for the prevention of nerve damage that leads to deformities and disabilities in leprosy patients.

## Materials and methods

### Recruitment of subjects

#### Patients and healthy controls

A total of 10 clinically confirmed leprosy patients in T1R and five healthy volunteers were recruited after taking informed consent from patients at The Leprosy Mission Community Hospital, New Delhi, India. The procedures for sample collection were in accordance with the standards and guidelines of the Indian Council of Medical Research. This study was approved by the “Ethical Committee-The Leprosy Mission Trust India (TLMTI),” in a meeting held on September 17, 2012. Demographic details of patients are given below (Table 1).

**TABLE 1:** Demographic characteristics of leprosy patients and healthy controls.

Characteristics		Type 1 reaction (T1R) (*n* = 10)	Healthy controls (*n* = 05)
Age (mean ± SD)		35 ± 12.12	32.65 ± 11.02
Gender (%)	Male	08 (80%)	03 (60%)
	Female	02 (20%)	02 (40%)
Bacillary index(mean ± SD)		2.36 ± 1.39	
Duration of disease		0–1 month	

#### Experimental animals

Outbred female New Zealand white rabbits were obtained from the Laboratory Animal Division of the Central Drug Research Institute (CDRI), Lucknow, and were maintained under standard conditions in the Department of Animal Experimentation of NJIL and OMD, ICMR, Agra. All animal experiments were approved by the Institutional Animal Ethical Committee and followed guidelines by the Animal Research Ethics Board of NJIL and OMD.

#### Production of polyclonal rabbit antibodies against *Mycobacterium leprae* sonicated antigen

To produce polyclonal antibodies rabbits were hyperimmunized with 250 μg of protein concentration of *M. leprae* soluble antigen (MLSA). The proteins were emulsified in Freund’s Incomplete Adjuvant (IFA) (Merck, Germany) and a 0.2 ml emulsion was injected intradermally at weekly intervals up to the 8th week. Blood samples (5 ml each time) were drawn from the marginal ear vein before each booster dose to check the level of antibody against MLSA. After 1 week of the 8th booster dose, blood was drawn from the marginal ear vein, and purification of IgG was carried out by the ammonium sulphate method as described earlier (Page and Thorpe, 2002).

### Sample collection

Slit skin scrapings (SSS) and saliva samples were collected from each subject. SSS samples were collected by taking four scrapes of tissue from an incision (5 mm long and 2 mm deep) made with the help of a sterile surgical scalpel blade on the left and right earlobes and skin lesions without any contamination with blood along the skin-slit area. The SSSs were collected in RNALater (Merck, Germany) and stored at −20°C until further use. Whole unstimulated saliva was collected into a pre-chilled, sterile 15 ml falcon tube through a sterile, pre-chilled funnel on ice and kept on ice throughout the procedure. Thereafter, Sterile Milli Q water was added to saliva samples (1:1 v/v) and vortexed to reduce the viscosity of the samples. The mixture was then centrifuged at 4,500 rpm for 15 min at 4°C. The supernatant was collected and stored at −80°C after adding a complete Protease Inhibitor Cocktail (Merck, Germany).

### Protein precipitation

The saliva samples were processed for protein precipitation by the TCA-Acetone-DTT method (Jessie et al., 2008). In brief, an equal volume of the mixture [TCA (20% v/v)-Acetone (80% v/v)-DTT (20 mm)] was added to pre-treated 500 μl of saliva, then vortexed to mix thoroughly, and allowed to precipitate overnight at −20°C. The mixture was then centrifuged at 15,000 rpm for 30 min at 4°C. The pellet thus obtained was washed with chilled acetone (90% v/v, 20 mm DTT) followed by acetone (80% v/v, 10 mm DTT). The dried pellet was resuspended in rehydration buffer (BioRad, USA) and stored at −80°C.

Protein precipitation from the SSS collected was done using the method described earlier (Bisht et al., 2006). In brief, 10 samples were pooled and centrifuged at 12,000 rpm for 10 min at 4°C, then the supernatant was decanted. Thereafter, an appropriate volume (1–2 ml) of sonication buffer (50 mm Tris*-*HCl containing 10 mm MgCl_2_, 0.1% sodium azide, 1 mm PMSF, and 1 mm EGTA; pH 7.4) was added before intermittent sonication for 15–20 min at 4°C. The homogenate was centrifuged at 12,000 rpm for 10–20 min at 4°C then the supernatant was collected and stored at −20°C until used. Further supernatant was treated with 1% SDS and then subjected to the TCA*-*acetone precipitation procedure. A 10% TCA (v/v) was added to the cell lysate drop by drop then the mixture was incubated at −20°C overnight and the precipitated protein was collected by centrifugation at 12,000 rpm for 15 min at 4°C. The pellet was washed with 90% ice-cold acetone and again with 100% ice-cold acetone and allowed to air dry. The protein pellets obtained were dissolved in an appropriate volume of two-dimensional (2D) rehydration buffer and the protein concentration was estimated using the Bradford method.

### Identification of cross-reactive proteins

#### Two-dimensional PAGE and isoelectric focusing

Two-dimensional (2D) PAGE and isoelectric focusing (IEF) were performed in two sets with proteins precipitated from saliva and SSS according to the method described earlier (Görg et al., 2000). In brief, 100 μg of protein samples were used to impregnate ReadyStrip IPG, 7 cm, pH 4–7 (Bio-Rad Laboratories, USA). Followed by overnight incubation for rehydration at 20°C, IEF was performed. After completion of IEF, IPG equilibration was performed to solubilise the focussed proteins and allow the SDS binding required for separation in the second dimension. The proteins were resolved in the second dimension in 12% SDS-PAGE. Thereafter western blotting was performed according to the previously described procedure (Towbin et al., 1979).

#### Western blotting

In brief, the resolving gel was removed from the electrophoresis system, and a blotting sandwich was assembled and allowed to transfer the proteins to nitrocellulose membrane (NCM) at 100 V for 1.5 h. After protein transfer, the blot was washed briefly with phosphate buffer saline (PBS) and blocking was performed by incubation with 2% BSA at 4°C overnight. The blot was washed with PBS after incubation and then incubated with primary antibody (Purified IgG from MLSA-hyperimmunized rabbit sera) in 100x dilution, for 2 h at RT. Thereafter, followed by washing with PBS containing 0.05% Tween-20 (PBS-T), the blot was later incubated with HRP conjugated anti-rabbit IgG (1000x dilution) for 1 h at RT. After that NCM was washed with PBS-T and reactive spots were developed by using substrate 3, 3’-diaminobenzidine (DAB) with hydrogen peroxide (H_2_O_2_). The image of the gel was captured by Chemidoc (Bio-Rad, USA). The spots of antigen-antibody reactivity on NCM were compared and identified in the second set of gel stained with Coomassie brilliant blue R-250. The software PDQuest (Bio-Rad, USA) was used to analyse 2D blot data. The 2D gel electrophoresis and western blotting were performed in duplicate two times to find out cross-reactive proteins. The spots of interest were excised and further analysed by mass spectrometry.

#### Mass spectrometry

MALDI-TOF was employed to identify the proteins which showed reactivity. In-gel, digestions of spots were done according to the protocol reported by Shevchenko et al. (1996). The procedure was adopted from our earlier study (Singh et al., 2015, 2018). In brief, an automated robotic enzymatic (Trypsin) digestion of spots of interest was done by using a protein digester (Model Investigator ProPrep, Genomic Solutions Ltd., UK) and was further purified by using ZipTipC18 (Millipore). The purified proteins were then applied to AnchorChip (Bruker) with 2 μl of the matrix [a saturated solution of α-cyano-4-hydroxycinnamic acid (HCCA) made in 50% ACN and 0.2% TFA]. Mass spectra of digested peptides were acquired by Autoflex II TOF/TOF50 (Bruker Daltonik GmgH, Leipzig, Germany) in positive reflectron mode and the detection range of 500–3,000 m/z. The peptide mass fingerprints were searched by using the Mascot Wizard program (Matrix Science, Ltd., London, United Kingdom). Search parameters used in MS/MS for the identification of spots were peptide mass tolerance ±0.5 Da ppm, peptide charge state 1+, and maximum missed cleavages one.

### Bioinformatic analysis

#### Validation of cross-reactivity

The cross-reactive host proteins identified were further validated by using bioinformatic tools. The peptide sequences of cross-reactive host proteins were analysed for homologous *M. leprae* proteins by using NCBI blastp (protein-protein BLAST)^1^.

#### Prediction of B cell epitopes in cross-reactive host proteins

The peptide sequences of cross-reactive host proteins were further analysed to predict BCEs. Based on the physio-chemical properties linear BCEs were predicted by submitting the sequence to BCPRED Server 1.0 (Saha and Raghava, 2004).

#### Three-dimensional structure of cross-reactive host proteins

The three-dimensional (3D) structures of cross-reactive proteins were deduced by submitting the sequence to the Phyre2 server (Kelley et al., 2015). Thereafter, the predicted BCEs were represented in 3D graphics by using the software VMD (1.9.4) (Humphrey et al., 1996).

## Results

### Cross-reactive salivary proteins

**FIGURE 1:**
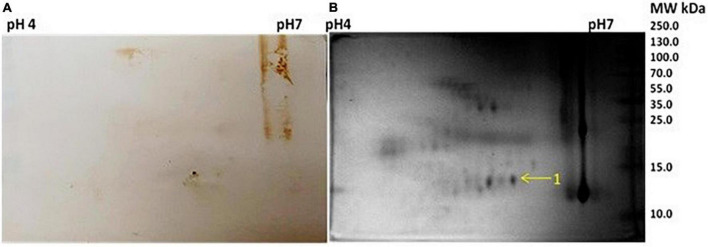
**(A)** Western blot picture with anti *Mycobacterium leprae* IgG of 2-dimensional (2D) electrophoresed saliva. **(B)** 2D gel electrophoresis profile of saliva.

**FIGURE 2:**
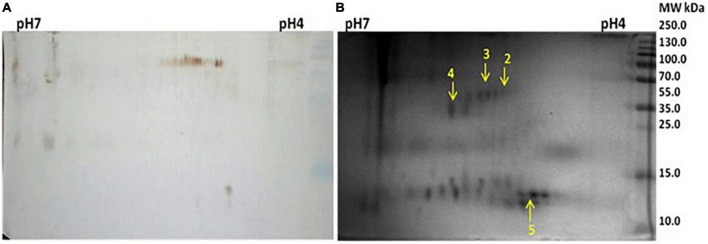
**(A)** Western blot against anti *Mycobacterium leprae* IgG of 2D electrophoresed saliva. **(B)** 2D gel electrophoresis profile of saliva.

As described earlier in the methodology section, the 2D gel electrophoresis, as well as western blotting, were performed with each protein sample extracted from saliva. Three samples showed reactivity when a western blot was performed with purified IgG from MLSA–hyperimmunized rabbit sera. We did not find any cross-reactive spots in the saliva and SSS samples of healthy controls. The cross-reactivities with clinical samples of T1R leprosy patients were identified as very light spots in some of the reactive sites; hence, the respective spots were marked by an arrow in the 2D gel electrophoresis images. The 2D gel images and respective blots are given below (Figures 1, 2).

Five spots were selected based on reactivity and search results of peptide mass fingerprints using the Mascot Wizard program are summarised in the table given below (Table 2).

**TABLE 2:** The protein spots identified by MS/MS.

Sr. no.	Spot no.	Name of protein	Protein source	Mascot score	MW (Da)	*E*-value
1	1	Protein S100-A9	Homo sapiens	75	13291	0.0089
2	2	Unnamed protein product, partial	Homo sapiens	88	35322	0.00049
3	3	Serpin peptidase inhibitor, clade A (alpha-1 antiproteinase, antitrypsin), member 1	Homo sapiens	156	46864	7.80E-11
4	4	Unnamed protein product, partial	Homo sapiens	68	41593	0.047
5	5	Cystatin SA-III	Homo sapiens	96	14409	6.90E-05

The Mascot Wizard program was used to search the database of Actinobacteria, Mycobacteria complex, and *Homo sapiens*. We found that all the identified reactive proteins were of *H. sapiens* in origin. S100-A9 protein of 13.2 kDa was identified at spot no. 1. Spot 2 and 4 were identified as 35.3 and 41.5 kDa proteins, respectively. Serpin peptidase inhibitor, clade A (Spot no. 3), and Cystatin SA-III (Spot no. 5) were also identified as cross-reactive.

### Cross-reactive proteins of slit skin scrapings

**FIGURE 3:**
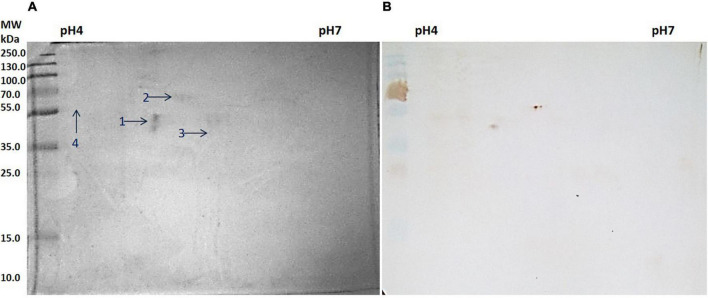
**(A)** Two-dimensional (2D) gel electrophoresis profile of slit skin scrapings (SSS). **(B)** Western blot against anti *Mycobacterium leprae* IgG.

Similar to salivary proteins, the 2D gel electrophoresis, as well as western blotting, were performed with protein samples extracted from pooled SSS. The 2D gel image and respective blot are given below. The spots of reactivity are marked in the respective 2D gel (Figure 3).

Four spots were selected based on reactivity and search results of peptide mass fingerprints using the Mascot Wizard program, which are summarised in the table given below (Table 3).

**TABLE 3:** The protein spots identified by MS/MS.

Sr no.	Spot no.	Name of protein	Protein source	Mascot score	MW (Da)	*E*-value
1	1	Unnamed protein product (partial)	Homo sapiens	102	41422	2.00E-05
2	2	Alpha-1 antitrypsin (AAT) variant	Homo sapiens	118	46577	5.00E-07
3	3	Vimentin, partial	Homo sapiens	99	35032	3.90E-05
4	4	Keratin 1	Homo sapiens	205	66029	9.90E-16

All four spots identified were of *H. sapiens* in origin. Spot number 1 was identified as a 41.4 kDa unnamed protein. Proteins from spot numbers 2, 3, and 4 were alpha-1 antitrypsin (AAT) variant, vimentin partial, and keratin 1, respectively.

### Validation of cross-reactivity by identifying host mimicking *Mycobacterium leprae* proteins

The identified cross-reactive host proteins were searched for homologous *M. leprae* proteins by using NCBI blastp for validation of the findings of 2D gel electrophoresis and western blotting. The results are summarised in the tables given below (Tables 4, 5).

**TABLE 4:** The mimicking salivary proteins of the host homologous to *Mycobacterium leprae* proteins.

Saliva spot no.	Host protein	Mimicking *M. leprae* protein (NCBI blastp)	Maximum score	*E* value	Accession number
1	Protein S100-A9	Hypothetical protein DIJ64_07765 [*Mycobacterium leprae*]	22.3	8.4	AWV48000.1
2	Unnamed protein product, partial	Carboxylic acid reductase [*Mycobacterium leprae*]	26.2	1.7	WP_041322427.1
3	Serpin peptidase inhibitor, clade A (alpha-1 antiproteinase, antitrypsin), member 1	AarF/ABC1/UbiB kinase family protein [*Mycobacterium leprae*]	31.2	0.062	WP_041323706.1
4	Unnamed protein product	AarF/ABC1/UbiB kinase family protein [*Mycobacterium leprae*]	28.9	0.28	WP_041323706.1
5	Cystatin SA-III	GTP cyclohydrolase I FolE [*Mycobacterium leprae*]	25.4	0.49	WP_010907607.1

**TABLE 5:** The mimicking slit skin scrapings proteins of the host homologous to *Mycobacterium leprae* proteins.

SSS spot no.	Host protein	Mimicking *M. leprae* protein (NCBI blastp)	Maximum score	*E* value	Accession number
1	Unnamed protein product, partial	AarF/ABC1/UbiB kinase family protein [*Mycobacterium leprae*]	28.9	0.51	WP_041323706.1
2	Alpha-1 antitrypsin variant	AarF/ABC1/UbiB kinase family protein [*Mycobacterium leprae*]	31.2	0.11	WP_041323706.1
3	Vimentin, partial	MMPL family transporter [*Mycobacterium leprae*]	25.4	5.3	AWV48827.1
4	Keratin 1	DivIVA domain-containing protein [*Mycobacterium leprae*]	33.1	0.036	WP_041323873.1

We found all the cross-reactive spots of saliva and SSS were showing sequence similarity to proteins of *M. leprae*. Spot 1 of saliva showed similarity with a hypothetical protein, DIJ64_07765. Furthermore, spots 2, 3, and 4 of saliva and spots 1 and 2 of SSS are either partial or variant of the same protein, i.e., AAT, and showed sequence similarity with an AarF/ABC1/UbiB kinase family protein. Spot 3 and 4 of SSS showed similarities with the mycobacterial membrane protein large (MMPL) family transporter protein and DivIVA domain-containing protein, respectively.

### B cell epitope prediction of cross-reactive host proteins

The linear BCEs were predicted for each host protein spot which were cross-reactive. Out of five spots of saliva samples only three spots, i.e., spots 2, 3, and 4 were showing the presence of BCEs. Since spots 2, 3, and 4 of saliva and spot 1 and 2 of SSS are partial/variants of the AAT, a total of 10 BCEs were considered for all these five spots. Furthermore, 5 and 7 BCEs were predicted for spots 3 and 4 of SSS, respectively. Based on the presence of BCEs in protein sequence, the spots were subsequently represented in 3D structures.

### Representation of B cell epitopes in the three-dimensional structure of cross-reactive proteins

**FIGURE 4:**
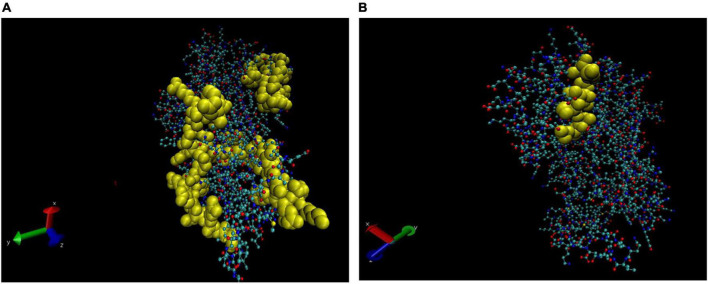
Three-dimensional (3D) structure of alpha-1 antitrypsin (AAT). The B cell epitopes (BCEs) are highlighted in yellow **(A,B)**.

**FIGURE 5:**
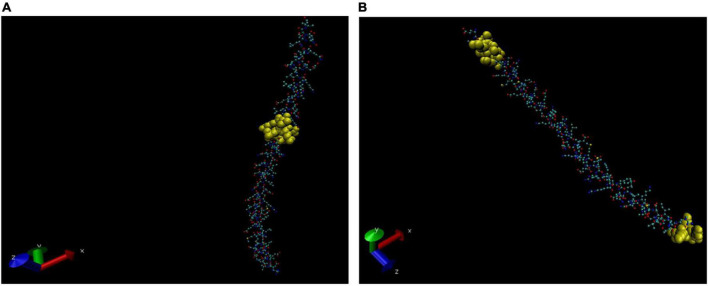
Three-dimensional (3D) structure of Vimentin (partial). The BCEs are highlighted in yellow **(A,B)**.

**FIGURE 6:**
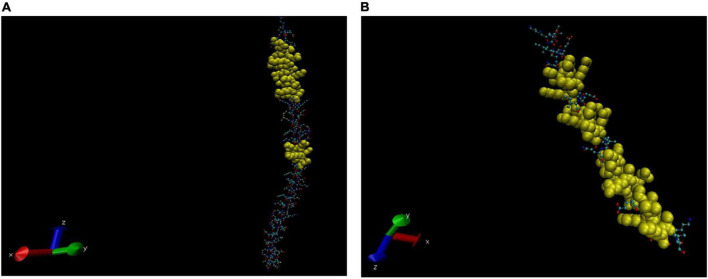
Three-dimensional (3D) structure of Keratin 1. The BCEs are highlighted in yellow **(A,B)**.

The 3D structures of cross-reactive host proteins were acquired in the PDB (Protein Data Bank) formatted model by using the Phyre2 server. The PDB files thus obtained were uploaded to software VMD (1.9.4) to visualise the structures. Thereafter, the BCEs of respective proteins were visualised by highlighting the sequence in the 3D structures. Depending on the sequence coverage by the Phyre2 server, the BCEs are shown in different images of the same protein (Figures 4–6).

## Discussion

Molecular mimicry plays an important role in autoimmune diseases, and it was initially indicated after finding cross-reactivity of the phosphoprotein of the measles virus and a protein of the herpes simplex virus type 1 with an intermediate filament protein (vimentin) of human cells. Several other cross-reactivities between viral proteins and host antigenic determinants have been subsequently reported (Fujinami et al., 1983). Several host proteins have previously been r reported to have an antigenic similarity and molecular mimicry and were found in concordance with disease progression (Singh et al., 2015, 2018).

In the present study, we attempted to find cross-reactivity between proteins of the host (leprosy patients) and pathogen (*M. leprae*) in two different types of clinical samples using 2-dimensional gel electrophoresis. The salivary, as well as SSS proteins, of leprosy patients in T1R, were allowed to react with polyclonal rabbit antibodies that were hyperimmunized with MLSA. Based on the results of western blotting, the cross-reactivity was found with five salivary proteins and four skin proteins. Among the five salivary proteins, that were found to be cross-reactive (Table 2), some of them might be interesting to explore through extensive studies to understand their role in the progression of the disease. The protein S100A9 (spot no. 1), a calcium-binding protein of the S100 family, is reported to be expressed abundantly in neutrophils and plays a prominent role in the regulation of inflammatory processes and immune response. It can induce neutrophil extravasation and macrophage activation, which can increase the bactericidal activity of neutrophils by promoting phagocytosis *via* activation of SYK, PI3K/AKT, and ERK1/2, and can induce degranulation of neutrophils by a MAPK-dependent mechanism. Yoshioka et al. (2016) have demonstrated the critical role of neutrophils and the S100A9 protein in granuloma formation, S100A9 function inhibition with specific inhibitor results in the impaired formation of organised granulomas with neutrophil cores. Granuloma formation is a very important defence mechanism to combat disease progression in leprosy. The pathogenesis of reactions in leprosy is characterised by inflammatory episodes and the protein S100A9 may be studied extensively to find a correlation between the levels of this protein and the progression of the T1R in leprosy.

Two proteins, i.e., 35.3 and 41.6 kDa (Spots 2 and 4) were identified as an unnamed protein product (partial), in saliva samples and were cross-reactive. The characterisation of these proteins may reveal their relevance in leprosy. An elevated level of AAT in LL and ENL has been reported in earlier studies (Yemul et al., 1983). We identified AAT in salivary cross-reactive protein at spot no. 3. Increased levels of AAT have been correlated with a high bacterial load, leading to the release of various proteases and the formation of immune complexes in ENL (Sengupta et al., 1983). The protein salivary cystatin SA-III is a potential precursor of the acquired enamel pellicle; however, its relevance in leprosy has not yet been reported.

Bioinformatic analysis reveals that spots 2, 3, and 4 of saliva are either partial or variants of the same protein, i.e., AAT, and mimic an *M. leprae* protein AarF/ABC1/UbiB kinase family protein. All the spots were searched for sequence similarity with *M. leprae* proteins, and the results are summarised in Table 4. All five spots of saliva were analysed for the prediction of BCEs, but only spots 2, 3, and 4 which correspond to AAT showed the presence of BCEs.

In the case of slit skin scraping, we found four cross-reactive proteins (Table 3). Spot no. 1 was identified as 41.4 kDa protein. AAT was also found to be cross-reactive in SSS (Spot no. 2). AAT is an anti-inflammatory protein with a well-known safety profile. The therapeutic potential of AAT has been tested in several autoimmune disease models. However, insufficiency or deficiency of AAT may be due to several reasons, but molecular mimicry or autoimmunity to self-proteins in the case of leprosy, as reported in earlier studies, suggests the requirement of extensive study to understand the role of AAT in leprosy as well. Furthermore, these BCEs could be explored further to understand their relevance in the pathogenesis of reactions and as a diagnostic marker for reactions in leprosy.

In earlier studies, it has been reported that anti-*M. leprae* monoclonal antibodies cross-react with human nerve as well as skin components, and it has been suggested that this antigenic similarity may be responsible for the development of autoimmune clinical manifestations in leprosy (Naafs et al., 1990; van den Akker et al., 1992). Mycobacterial 65 kDa heat shock protein has been reported to share a carboxy-terminal epitope with human epidermal cytokeratin 1/2 (Rambukkana et al., 1992). Earlier, cross-reactivity between human lactoferrin and the 65 kDa protein of tuberculosis and leprosy bacilli has been reported which was suggestive of the autoimmune progression of the disease (Esaguy et al., 1991). In the present study, vimentin was identified at spot no. 3 as a cross-reactive protein in SSS. Auto-antibodies against the cytoskeleton intermediate filament protein vimentin have been reported earlier in lepromatous leprosy patients (Frey et al., 1988). Auto-antibodies against different intermediate filaments have also been reported in other diseases like polymyositis/dermatomyositis, systemic sclerosis, rheumatoid arthritis (Senécal et al., 1985), and systemic lupus erythematosus (Alcover et al., 1984). We have recently reported molecular mimicry between tropomyosin and the probable ATP-dependent Clp protease ATP-binding subunit of *M. leprae* and suggested that it might be responsible for “leprous myositis” and muscular weakness (Singh et al., 2018). In a study, differences in the expression of cytoskeleton proteins vimentin and smooth muscle actin in the context of fuso-cellular macrophage transformation were explained to be responsible for hyperchromic indurated nodules in histoid leprosy (Canuto et al., 2018). The role of vimentin in T1R could be explored in further studies.

In the present study, the intermediate filament protein vimentin mimicked the MMPL family transporter protein of *M. leprae* when blastp (NCBI) was performed. The MMPL proteins are well recognised for their role in founding the mycobacterial cell envelope. The primary role of the MMPL is to translocate complex, virulence-associated envelope lipids and siderophores across the plasma membrane to the periplasmic space. MMPL transporters represent a subclass of RND transporters (Resistance-Nodulation-Cell Division) permeases. These mycobacterial proteins apparently have conducive roles in the virulence and drug resistance of the pathogens.

The intermediate filament protein vimentin might play an important role in the pathogenesis of reactions in leprosy, and it can be further investigated to find out its role in muscle damage during the progression of the disease and that of its homologous MMPL family transporter protein in virulence and antimicrobial resistance. Furthermore, the predicted BCEs of vimentin may also be useful as a biomarker for the reactions in leprosy.

High levels of anti-MBP antibodies in leprosy patients across the spectrum and cross-reactivity between epitopes of human myelin A1 with 50S ribosomal L2 and lysyl tRNA synthetase of *M. leprae* have been reported (Singh et al., 2015). Singh et al. reported molecular mimicry between cytokeratin-10 of keratin (host) protein and 65 kDa HSP (groEL2) of *M. leprae* and seven BCE of cytokeratin-10 and HSP 65 were found to be similar. Elevated levels of antibodies against keratin in leprosy patients, when compared with healthy controls, with the highest level in T1R followed by LL, BL, ENL, and TT/BT, have already been reported. Furthermore, the level of anti-keratin antibodies was clinically correlated with the number of lesions present in leprosy patients (Singh et al., 2012). Similarly in the present study, we identified keratin 1 (spot 4) as a cross-reactive protein.

The protein Keratin 1 showed homology with *M. leprae* protein domain-containing protein when blastp was performed within these proteins. It has been reported that coiled-coil proteins such as DivIVA play an important role in mycobacterial cell elongation (Pickford et al., 2020). Furthermore, the BCEs of the host protein Keratin 1 may be useful to predict T1R in leprosy. Recently, our group has reported a significant association of eight mimicking BCEs and five mimicking TCEs between *M. leprae* and host components with the inflammatory episodes of T1R in leprosy (Pathak et al., 2021). Antibodies against keratin might be due to the autoimmune phenomena in leprosy patients, as the majority of leprosy lesions are manifested in the skin, and the occurrence of keratosis is not an uncommon feature, especially during T1R of tuberculoid leprosy (Thangaraj et al., 1988; Thankappan and Sulochana, 1991).

Although the study was conducted with small sample size, we convincingly identified the presence of five cross-reactive proteins in saliva and four cross-reactive proteins in SSS of T1R leprosy patients. Furthermore, we predicted a total of 22 BCEs which might be useful as a prognostic biomarker for T1R in leprosy.

## Conclusion

Altogether, these data provide strong evidence for molecular mimicry between the host and pathogen in leprosy. The autoimmune phenomenon may contribute to the development of disabilities and deformities during the disease progression. Extensive studies with cross-reactive proteins as well as BCEs may lead to the identification of novel early diagnostic markers for reactions in leprosy. Furthermore, it may focus some light on the pathogenesis of reactions in leprosy and their relevance to the disease.

## Data availability statement

The original contributions presented in this study are included in the article/Supplementary material, further inquiries can be directed to the corresponding authors.

## Ethics statement

The studies involving human participants were reviewed and approved by the Ethical Committee of The Leprosy Mission Trust India (TLMTI). The patients/participants provided their written informed consent to participate in this study. The animal study was reviewed and approved by the Animal Research Ethics Board of National JALMA Institute for Leprosy & OMD, Indian Council of Medical Research, Agra, India.

## Author contributions

US and IS: conceptualisation, supervision, and resources. VP: investigation, methodology, and writing original draft of the manuscript. US, IS, and SS: validation and analysis. All authors contributed to the article and approved the submitted version.

## Footnotes

1
https://blast.ncbi.nlm.nih.gov/Blast.cgi?PAGE=Proteins
