# Supplementary material for: Corroboration of cross-reactivity between Mycobacterium leprae and hosts’ salivary and cutaneous proteins: A hope for prognostic biomarkers for the pathogenesis of reactions in leprosy
Source: Front Microbiol. 2022 Dec 6;13:1075053. doi: 10.3389/fmicb.2022.1075053 (PMC9764389; doi:10.3389/fmicb.2022.1075053)
Supplement: Supplementary file 1 [file Table_1.DOCX]

| **Characteristics** |  | **Type 1 reaction (n 10)** | **Healthy Controls (n 05)** |
| --- | --- | --- | --- |
| **Age (Mean ± SD)** |  | 35±12.12 | 32.65±11.02 |
| **Gender (%)** | Male | 08 (80%) | 03 (60%) |
|  | Female | 02 (20%) | 02 (40%) |
| **Bacillary index**  **(Mean ± SD)** |  | 2.36±1.39 | - |
| **Duration of disease** |  | 0 – 1 month | - |
