# Supplementary material for: Corroboration of cross-reactivity between Mycobacterium leprae and hosts’ salivary and cutaneous proteins: A hope for prognostic biomarkers for the pathogenesis of reactions in leprosy
Source: Front Microbiol. 2022 Dec 6;13:1075053. doi: 10.3389/fmicb.2022.1075053 (PMC9764389; doi:10.3389/fmicb.2022.1075053)
Supplement: Supplementary file 2 [file Table_2.DOCX]

| **Sr. No.** | **Spot No.** | **Name of protein** | **Protein source** | **Mascot Score** | **MW (Da)** | **E- Value** |
| --- | --- | --- | --- | --- | --- | --- |
| **1** | **1** | Protein S100-A9 | Homo sapiens | 75 | 13291 | 0.0089 |
| **2** | **2** | Unnamed protein product, partial | Homo sapiens | 88 | 35322 | 0.00049 |
| **3** | **3** | Serpin peptidase inhibitor, clade A (alpha-1 antiproteinase, antitrypsin), member 1 | Homo sapiens | 156 | 46864 | 7.80E-11 |
| **4** | **4** | Unnamed protein product, partial | Homo sapiens | 68 | 41593 | 0.047 |
| **5** | **5** | Cystatin SA-III | Homo sapiens | 96 | 14409 | 6.90E-05 |
