# Supplementary material for: Corroboration of cross-reactivity between Mycobacterium leprae and hosts’ salivary and cutaneous proteins: A hope for prognostic biomarkers for the pathogenesis of reactions in leprosy
Source: Front Microbiol. 2022 Dec 6;13:1075053. doi: 10.3389/fmicb.2022.1075053 (PMC9764389; doi:10.3389/fmicb.2022.1075053)
Supplement: Supplementary file 3 [file Table_3.DOCX]

| **Sr No.** | **Spot No.** | **Name of protein** | **Protein source** | **Mascot Score** | **MW (Da)** | **E- Value** |
| --- | --- | --- | --- | --- | --- | --- |
| **1** | **1** | Unnamed protein product (Partial) | Homo sapiens | 102 | 41422 | 2.00E-05 |
| **2** | **2** | Alpha-1 antitrypsin variant | Homo sapiens | 118 | 46577 | 5.00E-07 |
| **3** | **3** | Vimentin, Partial | Homo sapiens | 99 | 35032 | 3.90E-05 |
| **4** | **4** | Keratin 1 | Homo sapiens | 205 | 66029 | 9.90E-16 |
