# Supplementary material for: Corroboration of cross-reactivity between Mycobacterium leprae and hosts’ salivary and cutaneous proteins: A hope for prognostic biomarkers for the pathogenesis of reactions in leprosy
Source: Front Microbiol. 2022 Dec 6;13:1075053. doi: 10.3389/fmicb.2022.1075053 (PMC9764389; doi:10.3389/fmicb.2022.1075053)
Supplement: Supplementary file 4 [file Table_4.DOCX]

| **Saliva Spot no.** | **Host protein** | **Mimicking *M. leprae* protein (NCBI blastp)** | **Maximum score** | **E value** | **Accession number** |
| --- | --- | --- | --- | --- | --- |
| **1** | Protein S100-A9 | Hypothetical protein DIJ64_07765 [Mycobacterium leprae] | 22.3 | 8.4 | AWV48000.1 |
| **2** | Unnamed protein product, partial | Carboxylic acid reductase [Mycobacterium leprae] | 26.2 | 1.7 | WP_041322427.1 |
| **3** | Serpin peptidase inhibitor, clade A (alpha-1 antiproteinase, antitrypsin), member 1 | AarF/ABC1/UbiB kinase family protein [Mycobacterium leprae] | 31.2 | 0.062 | WP_041323706.1 |
| **4** | Unnamed protein product | AarF/ABC1/UbiB kinase family protein [Mycobacterium leprae] | 28.9 | 0.28 | WP_041323706.1 |
| **5** | Cystatin SA-III | GTP cyclohydrolase I FolE [Mycobacterium leprae] | 25.4 | 0.49 | WP_010907607.1 |
