# Supplementary material for: Corroboration of cross-reactivity between Mycobacterium leprae and hosts’ salivary and cutaneous proteins: A hope for prognostic biomarkers for the pathogenesis of reactions in leprosy
Source: Front Microbiol. 2022 Dec 6;13:1075053. doi: 10.3389/fmicb.2022.1075053 (PMC9764389; doi:10.3389/fmicb.2022.1075053)
Supplement: Supplementary file 5 [file Table_5.DOCX]

| **SSS Spot no.** | **Host protein** | **Mimicking *M. leprae* protein (NCBI blastp)** | **Maximum score** | **E value** | **Accession number** |
| --- | --- | --- | --- | --- | --- |
| **1** | Unnamed protein product, partial | AarF/ABC1/UbiB kinase family protein [Mycobacterium leprae] | 28.9 | 0.51 | WP_041323706.1 |
| **2** | Alpha-1 antitrypsin variant | AarF/ABC1/UbiB kinase family protein [Mycobacterium leprae] | 31.2 | 0.11 | WP_041323706.1 |
| **3** | Vimentin, partial | MMPL family transporter [Mycobacterium leprae] | 25.4 | 5.3 | AWV48827.1 |
| **4** | Keratin 1 | DivIVA domain-containing protein [Mycobacterium leprae] | 33.1 | 0.036 | WP_041323873.1 |
